# Obstacle Detection and Avoidance System Based on Monocular Camera and Size Expansion Algorithm for UAVs

**DOI:** 10.3390/s17051061

**Published:** 2017-05-07

**Authors:** Abdulla Al-Kaff, Fernando García, David Martín, Arturo De La Escalera, José María Armingol

**Affiliations:** Intelligent Systems Lab, Universidad Carlos III de Madrid, Leganes, 28911 Madrid, Spain; fegarcia@ing.uc3m.es (F.G.); dmgomez@ing.uc3m.es (D.M.); escalera@ing.uc3m.es (A.D.L.E.); armingol@ing.uc3m.es (J.M.A.)

**Keywords:** obstacle detection, collision avoidance, size expansion, feature points, UAV, monocular vision

## Abstract

One of the most challenging problems in the domain of autonomous aerial vehicles is the designing of a robust real-time obstacle detection and avoidance system. This problem is complex, especially for the micro and small aerial vehicles, that is due to the Size, Weight and Power (SWaP) constraints. Therefore, using lightweight sensors (i.e., Digital camera) can be the best choice comparing with other sensors; such as laser or radar.For real-time applications, different works are based on stereo cameras in order to obtain a 3D model of the obstacles, or to estimate their depth. Instead, in this paper, a method that mimics the human behavior of detecting the collision state of the approaching obstacles using monocular camera is proposed. The key of the proposed algorithm is to analyze the size changes of the detected feature points, combined with the expansion ratios of the convex hull constructed around the detected feature points from consecutive frames. During the Aerial Vehicle (UAV) motion, the detection algorithm estimates the changes in the size of the area of the approaching obstacles. First, the method detects the feature points of the obstacles, then extracts the obstacles that have the probability of getting close toward the UAV. Secondly, by comparing the area ratio of the obstacle and the position of the UAV, the method decides if the detected obstacle may cause a collision. Finally, by estimating the obstacle 2D position in the image and combining with the tracked waypoints, the UAV performs the avoidance maneuver. The proposed algorithm was evaluated by performing real indoor and outdoor flights, and the obtained results show the accuracy of the proposed algorithm compared with other related works.

## 1. Introduction

During the last decade, with the developments in microelectronics and the increase of computing efficiency, the use of Unmanned Aerial Vehicles (UAVs) is no longer restricted to the military purposes only. Recently, with the advent of small and micro aerial vehicles (sUAVs and MAVs), the requirements of operations and applications in low altitudes are increased.

Due to their ability to operate in remote, dangerous and dull situations, sUAVs and MAVs especially helicopters and vertical take-off and landing (VTOL) rotor-craft systems are increasingly used in many applications; such as surveying and mapping, rescue operation in disasters [1], spatial information acquisition, data collection from inaccessible areas and geophysics exploration [2,3], cooperate in manipulation and transportation [4], buildings inspection [5,6] and navigation purposes [7,8].

Nowadays, with the current technology and the variety and complexity of the tasks, modern UAVs aim at higher levels of autonomy and performing flight stabilization. For the autonomous UAVs, the ability of detection and avoidance of obstacles with high level of accuracy is considered to be a challenging problem.

The difficulty appears because of the size of UAVs is getting smaller and thus, the weight is getting lighter. Therefore, taking into account these properties, sUAVs and MAVs have not the ability of carrying heavy sensors such as laser [9,10,11] or radar [12]. Hence, the suitable solution is to use the on-board cameras due to its advantage of lightweight and low power consumption.

In addition to its lightweight and the low power consumption, the cameras provide rich information of the environment. Therefore, they are considered as important sensors mounted on the small and micro UAVs.

In vision-based navigation systems, different approaches were presented to solve the problem of obstacle detection and avoidance. Approaches such as [13,14,15], built a 3D model of the obstacle in the environment. Other works calculate the depth (distance) of the obstacles, such as in [16,17]. A technique based on stereo cameras, in order to estimate the proximity of the obstacles, was introduced in [18]. At which, the system detects the size and the position of the obstacles based on the disparity images and the view angle. Furthermore, this technique calculates the relation of the size and the distance of the detected obstacle to the UAV. All these approaches have the disadvantage of the high cost in the computational time.

Whilst bio-inspired (insect, animal or human like) approaches estimate the presence of the obstacle efficiently, without calculating the 3D model, such as using optical flow [19,20,21] or perspective cues [7,22,23]. However, optical flow approaches cannot identify the forward movement, due to the aperture problem, thus frontal obstacles would provide only movement component normal to the detected edges in the image, not providing frontal movement information *per se*. Perspective cues approaches work well in the structured environments [24].

Detecting and avoiding frontal obstacles using monocular camera is considered a challenging problem because of the absence of the optical flow or the motion parallax. However, size expansion provides useful information for detecting the obstacles that are moving towards the UAV.

From the bio-inspired point of view, the human visual system has the ability to extract information correctly of the objects that are moving toward them [25]. In addition, Gibson illustrated the ability of the human visual system to identify the approaching of the objects related to the expansion of its size, by both eyes or even one eye [26].

**Figure 1:**
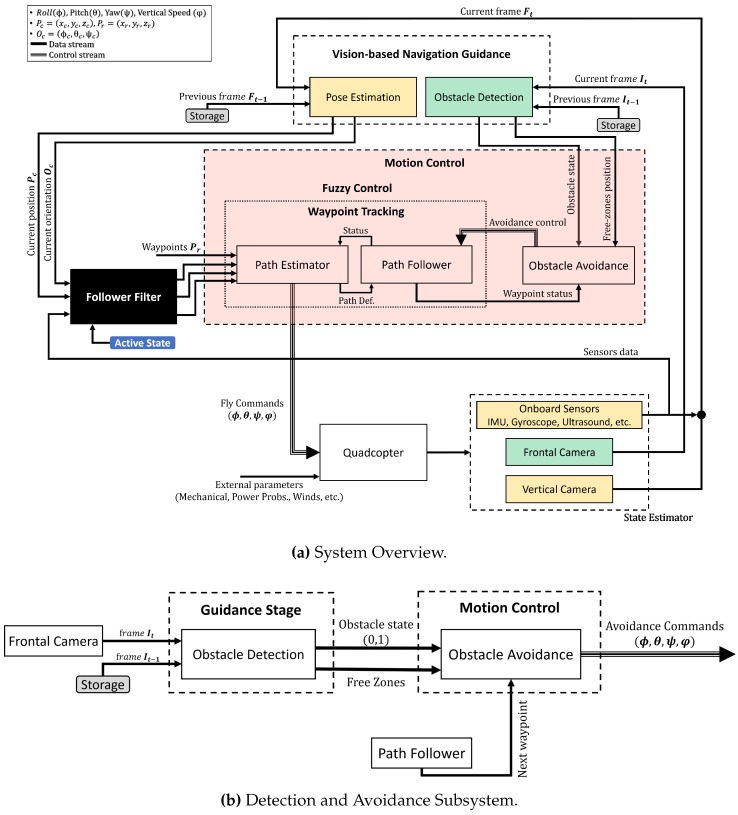
General overview of the Detection and Avoidance phases.

From this aspect, in this paper, a bio-inspired approach using a monocular camera is presented to mimic the human behavior of obstacle detection and avoidance applied on UAVs. The system is divided into two main stages: **Vision-Based Navigation and Guidance** in which, the obstacle detection algorithm is performed based on the input images captured from the front camera. In addition, **Motion Control**, where the avoidance decision is taken and sent to the UAV. Figure 1a shows the general overview of the system, whilst Figure 1b depicts the subsystem which focuses on the detection and avoidance stages.

**Figure 2:**
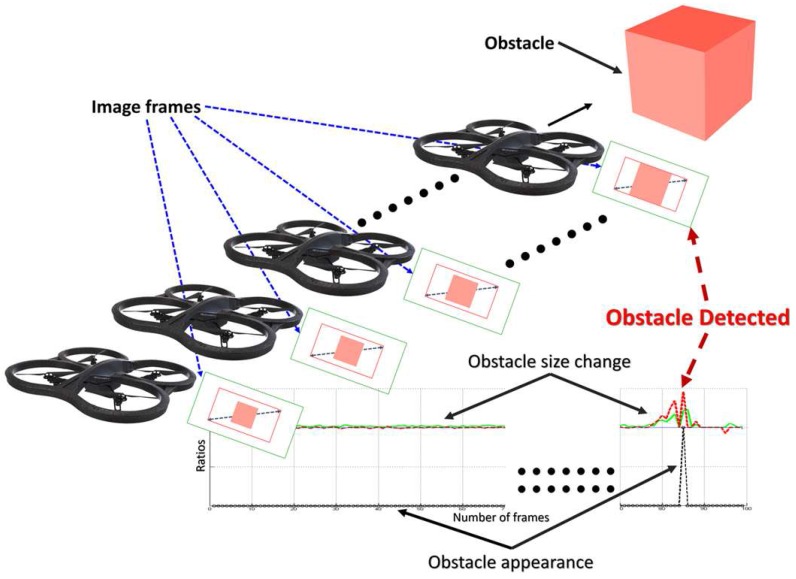
Concept of approaching obstacle detection.

The novelty in this paper is based on two main lines: First, the use of the size changes of the detected feature points, in order to provide object detection. Second, the changes in the size ratio in consecutive frames of the convex hull constructed from these points allow reliable frontal obstacle detection by means of a monocular camera and the motion of the UAV. The presence of approaching obstacles is estimated from these size expansion ratios, avoiding the need of complex 3D models, as shown in Figure 2. Reducing considerably the computation cost of the detection algorithm.

The remainder of this paper is organized as follows; Section 2 introduces the state-of-the-art work related to obstacle detection and avoidance approaches, followed by the proposed obstacle detection algorithm in Section 3. Section 4 presents the avoidance algorithm, then Section 5 discusses the experimental results. Finally, in Section 6 conclusion is summarized.

## 2. Related Work

Obstacle detection and avoidance plays an important role in any autonomous navigation system. Different works were presented to solve this challenging process especially in vision-based systems.

In [27], it was presented an approach based on the texture and color variation cue to detect obstacles for indoor environments. However, this approach works with detailed textures. Furthermore, their experiments were limited to indoor environments.

Working with Hybrid MAVs, Green et al. proposed an optical flow approach, mimicking the biological flying insects, by using dual cameras mounted on a fixed-wing UAV, in order to detect and avoid lateral obstacles [28]. Besides the detection of lateral obstacles only, some limitations appeared in avoiding large obstacles like walls. In addition, from the experiments, the avoidance algorithm is insufficient if the UAV flies in a straight path.

In [29], SIFT descriptor and Multi-scale Oriented-Patches (MOPS) are combined to show 3D information of the obstacles. At which, the edges and corners of the object are extracted using MOPS by obtaining and matching the MOPS feature points of the corners, then the 3D spatial information of the MOPS points is extracted. After that, SIFT is used to detect the internal outline information. However, the presented approach has expensive computational time (577 ms).

Bills et al. [7] proposed an approach for indoor environments with a uniform structure characteristics. In this work, Hough Transform is used to detect the edges that are used to classify the essence of the scene based on a trained classifier. However, their experiments were limited to corridors and stairs areas.

A saliency method based on Discrete Cosine Transform (DCT) is presented in [30] for obstacle detection purposes. From the input images, the system assumes that the obstacle is a unique content in a repeated redundant background, then by applying amplitude spectrum suppression, the method can remove the background. Finally, by using the Inverse Discrete Cosine Transform (IDCT) and a threshold algorithm, the center of the obstacle is obtained. Furthermore, a pinhole camera model is used to estimate the relative angle between the UAV and the obstacle, this angle is used with a PD controller to control the heading of the UAV for obstacle avoidance.

In [17], the authors presented an approach for measuring the relative distance to the obstacle. At which, the camera position is estimated based on the Extended Kalman Filter (EKF) and the IMU data. Then the 3D position of the obstacle can be calculated by back projecting the detected features of the obstacle from its images.

An expansion segmentation method was presented in [31], in which a conditional Markov Random Field (MRF) is used to distinguish if the frontal object may represent a collision or not. Additionally, an inertial system is used to estimate the collision time. However, the experiments of this work was limited to simulations.

Another approach presented in [24] used the feature detection algorithm in conjunction with the template matching to detect the size expansions of the obstacles. However, the experiments were limited to tree-like obstacles and did not show results of other shapes.

Kim et al. presented a block-based motion estimation approach for detecting the moving obstacles (humans) [32]. In which, the input image is divided into smaller blocks, then the system compare the motion in each block through consecutive images. However, their experiments were limited in detecting large size obstacles (humans) in indoor environments.

In addition, surveys of different approaches of UAVs guidance, navigation and collision avoidance methods and technologies are presented in [33,34]. Recently, Mcfadyen et al. presented a literature review of the vision-based collision avoidance systems [35].

The work presented in this paper represents a step forward in the obstacle detection and avoidance by the use of the convex hull (area) of the obstacle identified by means of image features. The approach is able to work in real time with all different kind of obstacles and in different tested scenarios.

## 3. Obstacle Detection

The proposed obstacle detection algorithm mimics the human behavior of detecting the obstacles that are located in front of the UAV during motion. At which point, the collision state of the approaching obstacles is estimated instead of building 3D models, or calculating the depth of the obstacle.

**Figure 3:**
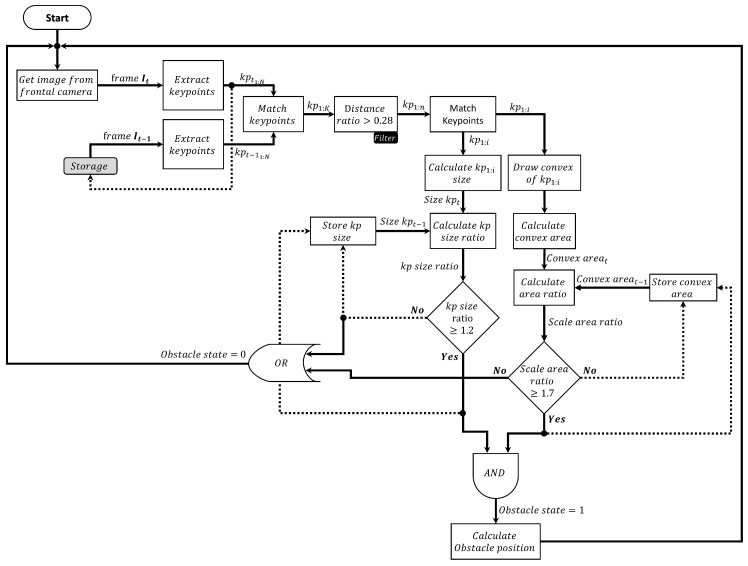
Obstacle detection approach flowchart.

The novelty and the key of this algorithm is to estimate the size ratios of the approaching obstacles from the consecutive frames during the flight as shown in Figure 2. This is achieved by estimating the change in the size property of the detected feature points (diameter), and the size of the convex hull (area) which is constructed from these points as well. When the size ratios exceed certain empirical values (explained in Section 3.2), it means that there is an obstacle detected, and can cause a danger to the UAV as shown in Algorithm 1, and Figure 3.
**Algorithm 1:** Obstacle Detection
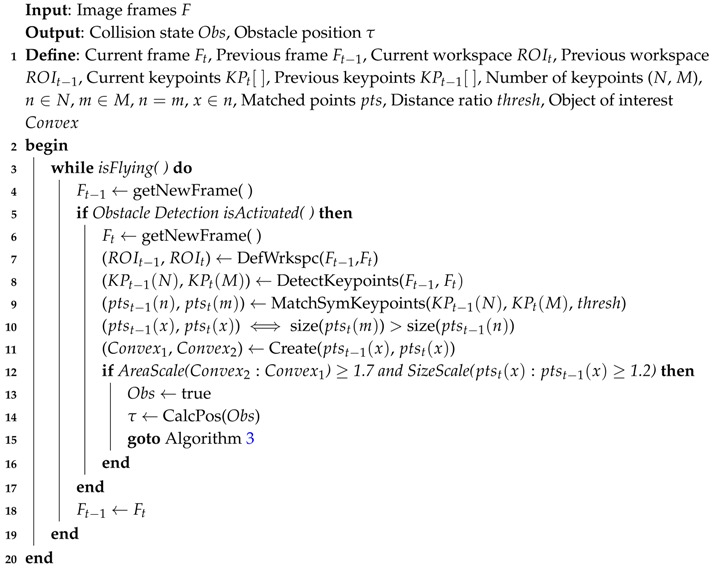


### 3.1. Feature Detection and Description

**Figure 4:**
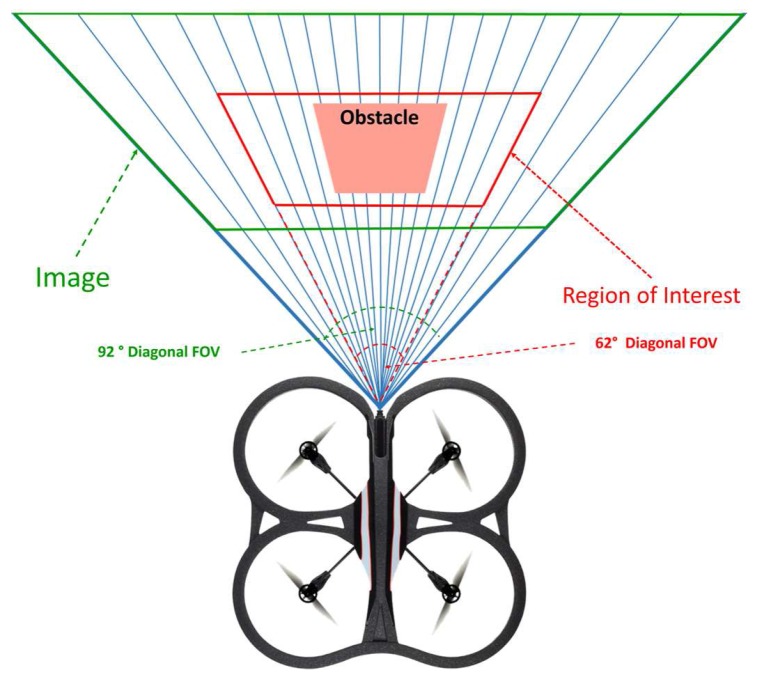
Define the diagonal $62^{\circ }$ patch from the whole $92^{\circ }$ image FOV.

In this step, an image Region Of Interest (ROI) of diagonal $62^{\circ }$ Field of View (FOV) is taken, in order to be processed instead of the whole image, as shown in Figure 4. The selection of the diagonal $62^{\circ }$ ROI is based on the results that are obtained from the experiments. Where, it has been found that any object detected out of the area of this ROI will not cause any danger to the UAV, and only the objects that are detected in the scope of this diagonal $62^{\circ }$ ROI can be considered as an obstacle. Furthermore, processing the diagonal $62^{\circ }$ ROI instead of the whole diagonal $92^{\circ }$ image, leads to a significant minimizing in computational time. Test performed proved the viability of this approach, and the results will be discussed in following sections.

Due to flying in unknown environments, the captured frames are affected by different conditions; such as illumination variation which may induce to noise and error. However, the keypoints need to be extracted accurately even under these conditions. Therefore the SIFT detector algorithm is used; because of the ability to identify and localize accurately the feature points even under different image condition specially scale and rotation properties.

**Figure 5:**
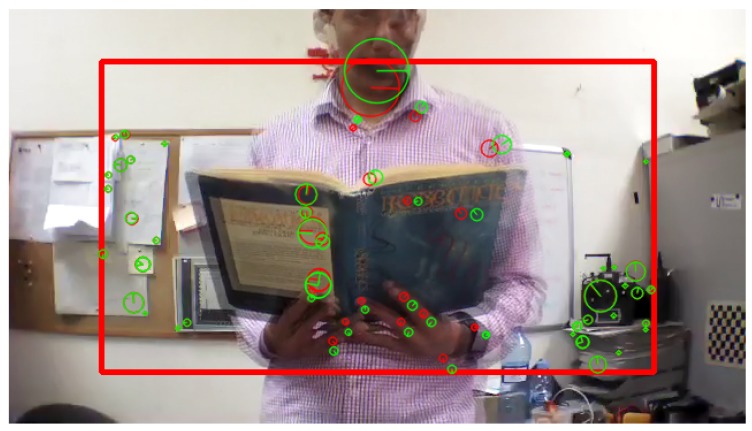
Keypoints extraction from two consecutive frames; keypoints extracted from frame $f_{t-1}$ (red) and keypoints extracted from frame $f_{t}$ (green).

According to Algorithm 1, all the keypoints are detected and its descriptors are extracted from the two consecutive frames as shown in Figure 5, then a vector of the position (x,y) and the size (s) of each keypoint is obtained.

After detection the keypoints, the Brute-Force algorithm is applied to match the keypoints from the two frames, and only the points that are found in both frames are returned. Algorithm 2 illustrates the concept of the Brute-Force method to match, and find the smallest distance of a pair of points.
**Algorithm 2:** Brute-Force Matcher
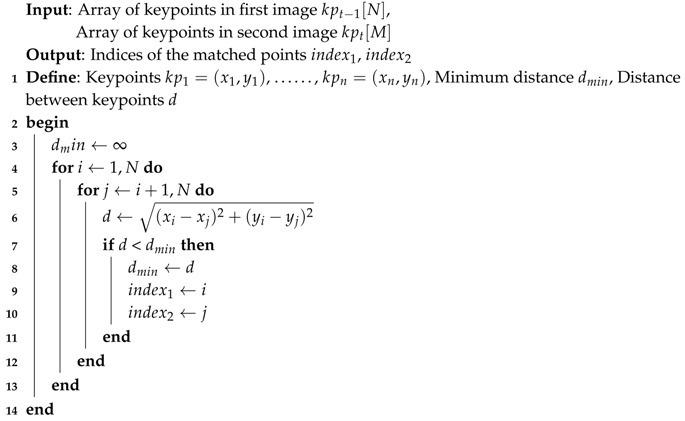


For more accuracy, the matched keypoints are filtered, by eliminating the ones that have a minimum distance ratio bigger than an empirical threshold value (0.28). Let mkp is the filtered-matched keypoint which are calculated as follows:

(1) $$mkp\left(n\right)=\left\{\begin{array}{cc} (x,y,s), & \mathrm{distratio}\leq 0.28\\ 0, & otherwise \end{array}\right.\;\;\forall n\in K$$

where, *s* is the size of the keypoint (diameter), distratio is the minimum distance ratio of the matched keypoints, and *K* is the total number of the matched keypoints.

**Figure 6:**
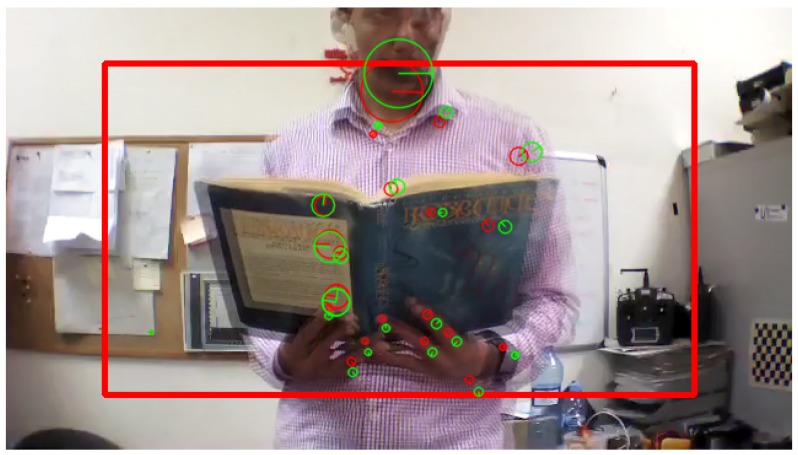
Filtered keypoints where the size expand from the second frame to the first frame; keypoints extracted from frame $f_{t-1}$ (red) and keypoints extracted from frame $f_{t}$ (green).

Afterwards, the obtained keypoints by Equation (1) are compared from the second to the first frame, and then the algorithm return the matched keypoints if and only if its size is growing, as shown in Figure 6:

(2) $$mkp\left(i\right)=\left\{\begin{array}{cc} (x,y,s), & Size(mkp_{2}\left(i\right)>mkp_{1}\left(i\right))\\ 0, & otherwise \end{array}\right.\;\;\forall i\in n$$

### 3.2. Object of Interest (OOI)

**Figure 7:**
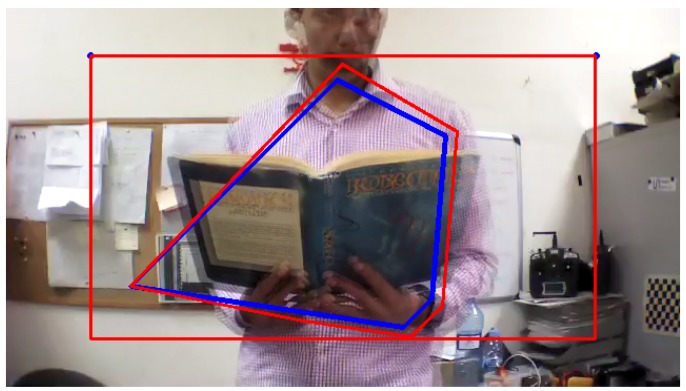
Convex Hull construction from detected keypoints in both frames; frame $f_{t-1}$ (blue) and $f_{t}$ (red).

The next step of the detection algorithm is to determine the probability to detect a frontal obstacle. Hence, from the extracted and filtered keypoints by Equation (2), an Object of Interest (OOI) is created around these keypoints in both frames, by creating a convex hull of the corresponding points, as it is shown in Figure 7:

(3) $$C=\sum_{i=1}^{N}\lambda_{i}mpk_{i}|\left(\forall i:\lambda_{i}\geq 0\right)$$

where *C* defines the convex hull, and $\lambda_{i}$ is a non-negative weight assigned to the keypoints $mpk_{i}\in N$ and $\sum_{i=1}^{N}\lambda_{i}=1$.

Next, in order to estimate the changes in the size of the area of the detected obstacles, it is considered that each convex hull as an irregular polygon. Therefore, for a given *C* as a convex hull, the area of *C* can be calculated as follows:

(4) $$\begin{array}{c} C_{area}=\frac{1}{2}\left|\begin{array}{cc} x_{1} & y_{1}\\ x_{2} & y_{2}\\ x_{3} & y_{3}\\ \vdots & \vdots \\ x_{n} & y_{n}\\ x_{1} & y_{1} \end{array}\right|=\frac{1}{2}\begin{array}{c} {}[\left(x_{1}y_{2}+x_{2}y_{3}+x_{3}y_{4}+\cdots +x_{n}y_{1}\right)\\ \;-(y_{1}x_{2}+y_{2}x_{3}+y_{3}x_{4}+\cdots +y_{n}x_{1})] \end{array} \end{array}$$

where $x_{\left(1:n\right)}$ and $y_{\left(1:n\right)}$ are vertices, and *n* is the number of sides of the polygon.

Finally, the size ratio of the matched keypoints, and the area of the convex hull from the second to the first frame are calculated respectively as follows:

(5) $$ratio\left(mkp\right)=\frac{1}{N}\sum_{i=1}^{N}\frac{Size(mkp_{2}\left(i\right))}{Size(mkp_{1}\left(i\right))}$$

(6) $$ratio\left(C\right)=\frac{Size(C_{2})}{Size(C_{1})}$$

Then, the algorithm estimates the collision state, if the approaching obstacle may represent a collision or not.

(7) $$State=\left\{\begin{array}{cc} 1, & ratio(mkp)\geq 1.2⋀ratio(C)\geq 1.7\\ 0, & otherwise \end{array}\right.$$

**Figure 8:**
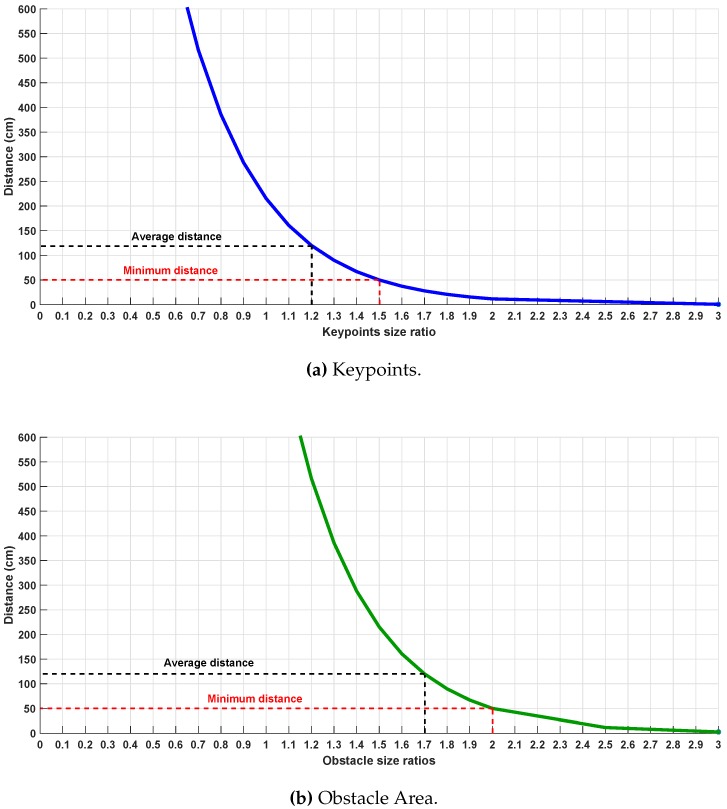
Distance and Ratios relation.

Next, an empirical study about the relation of the ratios between the size of the keypoints, the area of the obstacle and the distance of the approaching obstacle has been developed and the results are illustrated in Figure 8. This relation has been estimated by performing different indoor and outdoor experiments. Assuming that the UAV is flying at a constant velocity, the best ratios are in the range of [1.2–1.5], and [1.7–2.0] for keypoints size and obstacle size area respectively, at which the obstacle can be detected in a distance of [120–50] cm.

**Figure 9:**
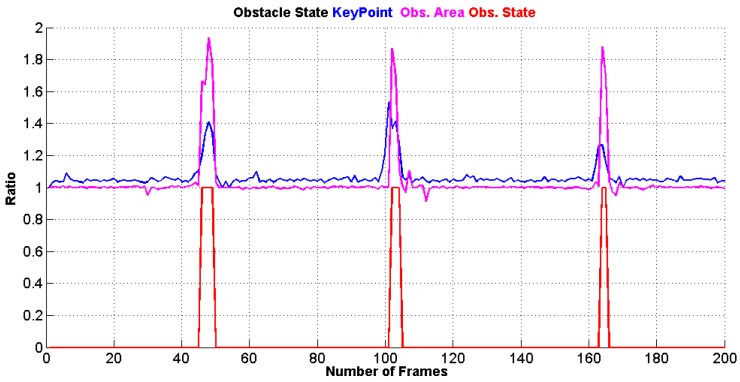
Obstacle State: Keypoint size ratio (Blue), Convex hull area ratio (Magenta) and Obstacle State **(0)** not fount **(1)** found (Red).

Figure 9 shows the collision state of the detected obstacles by the monocular camera, where it provides **1** if there is an obstacle, or it provides **0** if there is no obstacle detected.

**Figure 10:**
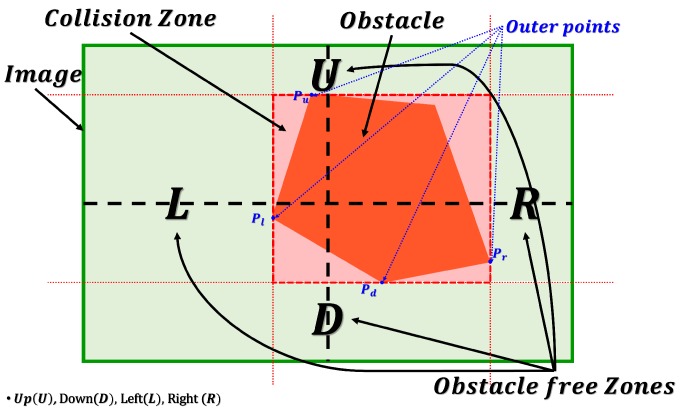
Estimating Obstacle outer points.

In this step, after detecting the obstacles with a collision state value 1, the algorithm estimates the position of the extremely outer points that construct the obstacle in the image $\left(P_{l},P_{r},P_{u},P_{d}\right)$, as it is shown in Figure 10, where $P_{l}$ is the point the of a position that has the minimum *x* value, $P_{r}$ has the maximum *x* value, and similarly, $P_{u}$ and $P_{d}$ have the *y* minimum and maximum values respectively.

Finally, the collision-free zones Left, Right, Up and Down (in case of hanged or flying obstacles) are calculated as four rectangles surrounding the obstacle as shown en Equation (8):

(8) $$\begin{array}{cc} \tau & =\left(\begin{array}{cccc} \tau_{l}, & \tau_{r}, & \tau_{u}, & \tau_{d} \end{array}\right)\\ & =\left(\begin{array}{cccc} Zone_{L_{width}}, & Zone_{R_{width}}, & Zone_{U_{height}}, & Zone_{D_{height}} \end{array}\right) \end{array}$$

where, $Zone_{L_{width}}$, $Zone_{R_{width}}$, $Zone_{U_{height}}$ and $Zone_{D_{height}}$ are the width and the height of the rectangles that are created by the points $\left(P_{l},P_{r},P_{u},P_{d}\right)$, as follows:

(9) $$\begin{array}{cc} Zone_{L} & =Rectangle\left[\begin{array}{cccc} (0,P_{u_{y}}), & (P_{l_{x}},P_{u_{y}}), & (0,P_{d_{y}}), & \left(P_{l_{x}},P_{d_{y}}\right) \end{array}\right]\\ Zone_{R} & =Rectangle\left[\begin{array}{cccc} (P_{r_{x}},P_{u_{y}}), & (ROI_{w},P_{u_{y}}), & (P_{r_{x}},P_{d_{y}}), & \left(ROI_{w},P_{d_{y}}\right) \end{array}\right]\\ Zone_{U} & =Rectangle\left[\begin{array}{cccc} (P_{l_{x}},0), & (P_{r_{x}},0), & (P_{l_{x}},P_{u_{y}}), & \left(P_{r_{x}},P_{u_{y}}\right) \end{array}\right]\\ Zone_{D} & =Rectangle\left[\begin{array}{cccc} (P_{l_{x}},P_{d_{y}}), & (P_{r_{x}},P_{d_{y}}), & (P_{l_{x}},ROI_{h}), & \left(P_{r_{x}},ROI_{h}\right) \end{array}\right] \end{array}$$

where, *w* and *h* are the width and the height of the ROI respectively.

## 4. Obstacle Avoidance

**Figure 11:**
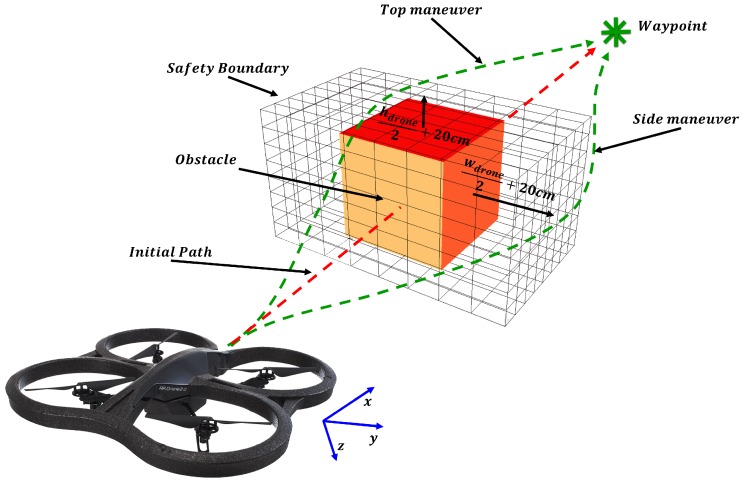
Obstacle Avoidance Path.

In this section, the combined mission of the waypoint tracking and the avoidance method is described. The geometrical problem is shown in Figure 11 where the avoidance technique is summarized in Algorithm 3.

To define the problem of waypoint tracking, let the UAV *X* flying at a velocity *V*, considering the UAV flies forward at a constant velocity along its *x-axis*, where:

(10) $$\begin{array}{cc} X & ={\left[\begin{array}{ccc} x_{d} & y_{d} & z_{d} \end{array}\right]}^{T}\;and,\\ V & ={\left[\begin{array}{ccc} u_{d} & v_{d} & w_{d} \end{array}\right]}^{T} \end{array}$$

On the other hand, let a waypoint:

(11) $$WP={\left[\begin{array}{ccc} x_{w} & y_{w} & z_{w} \end{array}\right]}^{T}$$

hence the waypoint is assumed to be tracked if $x_{d}$ is achieved when both $y_{d}$ and $z_{d}$ are satisfied, where:

(12) $$\begin{array}{cc} x_{d} & =x_{w}\pm \mu_{x}\\ y_{d} & =y_{w}\pm \mu_{y}\\ z_{d} & =z_{w}\pm \mu_{z} \end{array}$$

where, μ is the tolerance area around the waypoint position with a radius of 10 cm from the waypoint.

Let a frontal obstacle obs be detected by Algorithm 1, situated in the UAV path and surrounded by the collision-free zones $\tau =(\tau_{l},\tau_{r},\tau_{u},\tau_{d})$.

First, the avoidance algorithm checks all the free zones and differentiate which zone is the best to be followed. This is done by reading the position of the next waypoint and by comparing the size of the free zones, where the final maneuver will be in term of **Left-Right** or **Up-Down** motion or a combination of both. After that, a safety boundary surrounding the obstacle is assumed as shown in Figure 11, which is based on the dimensions of the UAV. This safety region is estimated to be:

(13) $$Safety_{lr}=\left(\frac{w_{UAV}}{2}\right)+20\;[\mathrm{cm}]$$

and

(14) $$Safety_{ud}=\left(\frac{h_{UAV}}{2}\right)+20\;[\mathrm{cm}]$$

where, *w* and *h* defines the width and the height of the UAV respectively.

Afterwards, the algorithm reads the position of the predefined next waypoint, and calculates the new waypoint out of the path (in order to avoid the obstacle), and sends a control command (velocity control) to the UAV for maneuvering according to the waypoint position as follows:

- Horizontal maneuver (*Right* or *Left*)
  (15) $$\;V_{lr}=\kappa \left(y_{d}\pm Safety_{lr}\right)$$
- Vertical maneuver (*Top* or *bottom*)
  (16) $$\;V_{ud}=\kappa \left(z_{d}\pm Safety_{ud}\right)$$

where $y_{d}$ and $z_{d}$ are the UAV position in (y,z)-coordinates, and κ is a control coefficient.
**Algorithm 3:** Obstacle Avoidance Algorithm
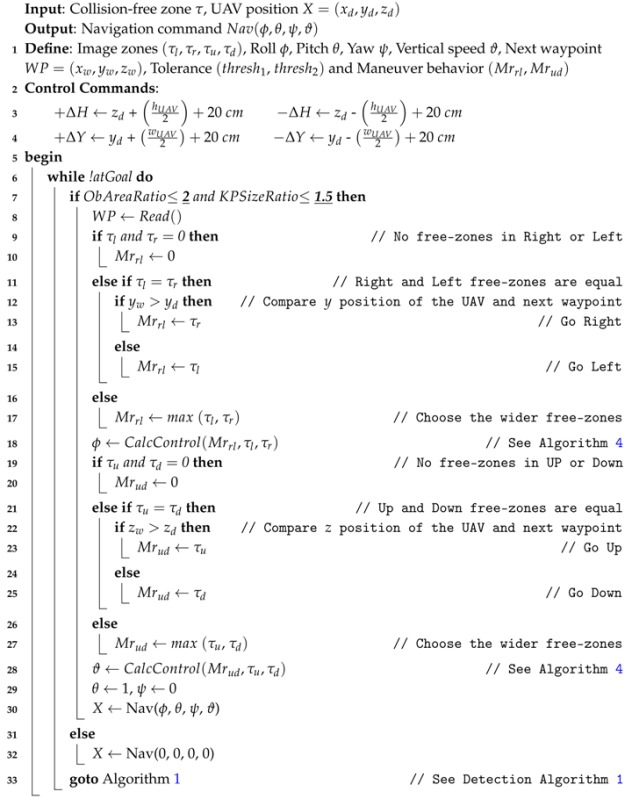

**Algorithm 4:** Calculate Avoidance Control
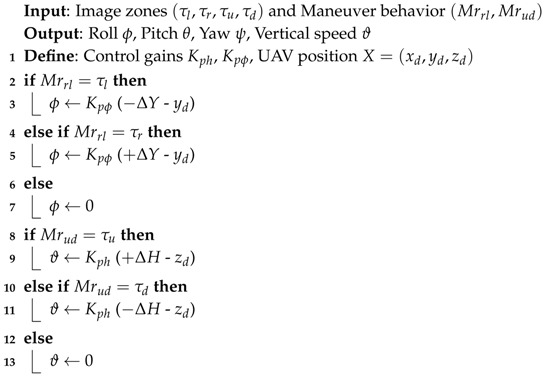


Finally, by estimating the new UAV position after avoidance, the algorithm recalculates the new waypoints in order the UAV to be able to return back to its predefined path and activate the detection process.

In the case that the AreaScale is greater than 2 and the SizeScale of the keypoints is greater than 1.5, a "Hover" command is sent to the UAV. That is because if the ratios exceed these limits, this means that the obstacle is very close to the UAV (less than 50 cm), as it is shown in Figure 8.

## 5. Experimental Results

In order to evaluate the performance of the proposed algorithms in the previous sections, 100 different real flight experiments have been carried out, in both indoor and outdoor environments, with a total number of 1000 obstacles, taking in consideration the visual conditions (the illumination and the texture of the obstacles) which affect the accuracy of the detection.

### 5.1. Platform

The processing in the ground station is performed in Intel i7-3770 at 3.4 GHz CPU, with 6 GB DDR3 RAM. The connection with the UAV is established via a standard 802.11n wireless LAN card.

The experiments have been performed with a Parrot AR.Drone 2.0 quadcopter [36]. This control system is governed by the inputs (roll, pitch, yaw angles, and vertical speed), therefore the implemented controller realize the UAV actual position, orientation and velocity.

One of the most important aspects in the avoidance phase is based on the robust control system for the UAV at which it is necessary to know its dynamic model. However, to avoid the complexity in modeling, the avoidance control was applied over the internal control of the system, modifying the roll and the vertical speed in order to perform the maneuvers in *y* and *z* directions.

### 5.2. Scenarios

Two different scenarios have been conducted, in order to evaluate the performance of the proposed algorithms in both motion and stability, with data gathered from the experiments to test the detection and the estimation of the position of the obstacle. In each scenario, different types of obstacles (people, obstacles, pillars, trees and walls), (static and dynamic) are situated.

The first scenario is a predefined straight flight, where the UAV flies in a straight line from the starting point to the end point. Different types of obstacles with unknown previous position were situated in the UAV path. The goal of this scenario is to evaluate the accuracy, and robustness in detecting, and avoiding the obstacles in motion.

The second scenario is a hover stability flight. At which, the UAV enters to the hover flight mode, and different obstacle are approaching to it. Once an obstacle is detected, the UAV flights in the opposite direction of the obstacle (Backward maneuver).

### 5.3. Results (Obstacle Detection)

From the experiments, the obtained results demonstrate that the algorithm is able to detect the obstacles with different sizes (areas) between 8500 and 200,000 pixels, and at a distance range between 90 and 120 cm. It is shown that the minimum accuracy of the algorithm is 95.0%, and the overall accuracy is 97.4% as it is demonstrated in Table 1.

**Figure 12:**
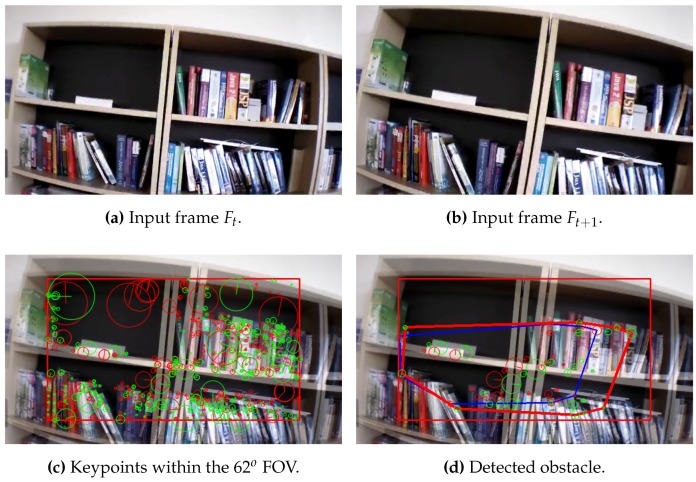
Obstacle detection: ratio(mkp) = 1.27, ratio(C) = 1.76 and distance = 114 cm.

**Figure 13:**
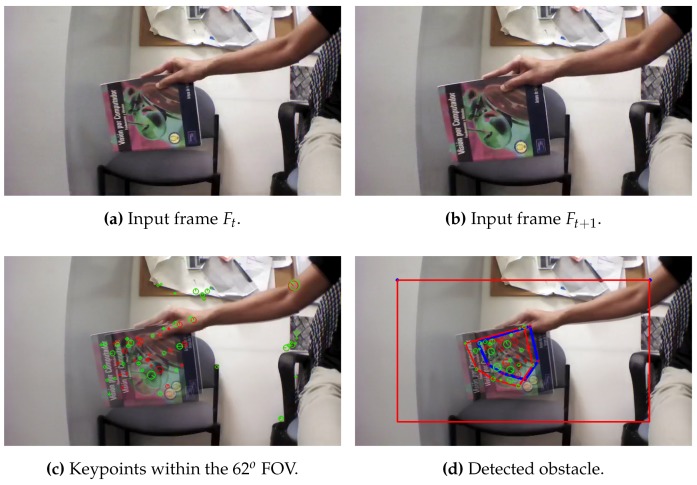
Obstacle detection: ratio(mkp) = 1.25, ratio(C) = 1.71 and distance = 92 cm.

**Figure 14:**
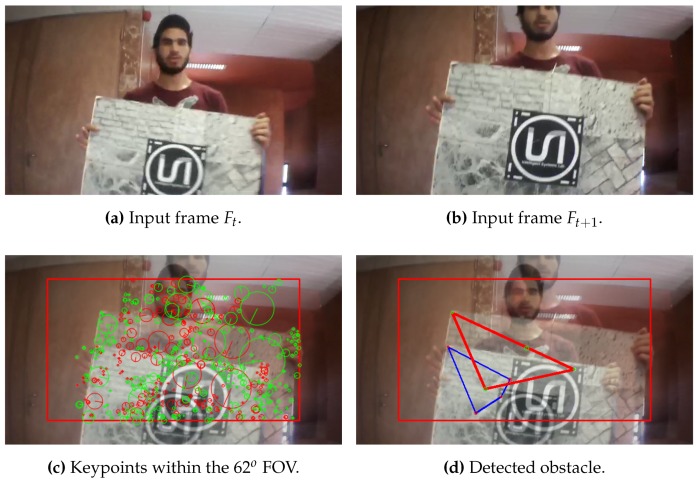
Obstacle detection: ratio(mkp) = 1.20, ratio(C) = 2.15 and distance = 126 cm.

Figure 12, Figure 13 and Figure 14 illustrate the detection process of various approaching obstacles, with different size ratios. Where, Figure 12a,b, Figure 13a,b and Figure 14a,b are showing the two input consecutive frames to be processed. In Figure 12c, Figure 13c or Figure 14c it is shown the total number of the detected and matched keypoints before filtering its size expansion property. Finally, the filtered keypoints and the constructed polygon of the detected obstacle are shown in Figure 12d, Figure 13d and Figure 14d.

Table 1 summarizes the accuracy of the detection algorithm. The table shows the total number of the obstacles that either situated in the UAV path (first scenario) or moving towards the UAV (second scenario), the number of the detected obstacles and the number of fails.

From the table, it is illustrated that the accuracy of the detection process in the indoor scenarios is better than the accuracy in outdoor environments. This is due to the constancy of the light conditions in indoors rather than outdoors, which are suffered from various lighting effects.

**Figure 15:**
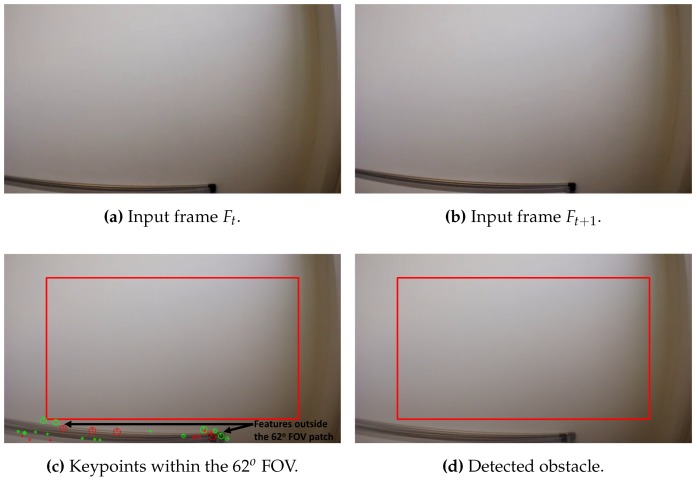
Obstacle detection fail (wall) (absence of texture): ratio(mkp) = 1 and ratio(C) = 1.

Two main reasons for the fail of detection; the first one is the disability of extracting sufficient number of keypoints, and that is either because of the low light conditions or because of the absence of the texture on the obstacle surface such as in the case of some pillars and walls as shown in Figure 15.

The second reason is the direction of the motion of the obstacle, the algorithm is able to detect the moving obstacle if the motion is towards the UAV.

**Figure 16:**
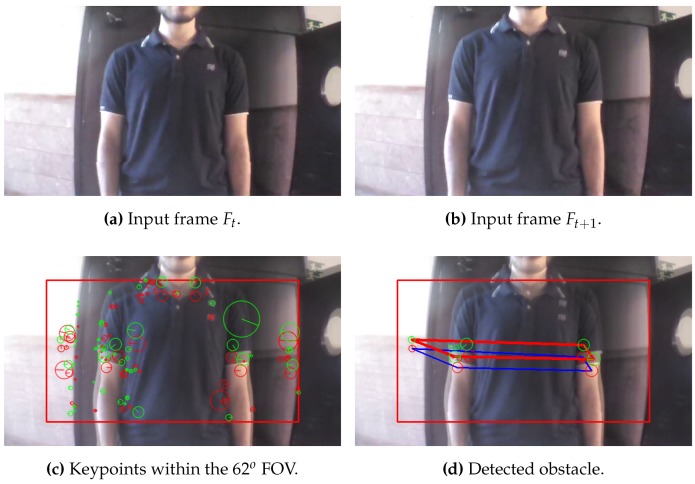
Obstacle detection fail (people) (motion around the UAV)-KPSizeRatio = 1.07 and ObAreaRatio = 1.03.

Figure 16 shows an example of the second scenario, where the UAV flies in hover mode, and the object is moving, however, this movement is not in the direction of the UAV. Therefore, it does not consider as an obstacle.

However, in most cases of the moving obstacles according to Table 1, the algorithm could not detect the appearance of the obstacles if the motion is around the UAV such as in the case of the people and obstacles.

In addition, the proposed algorithm is evaluated against two related works of detecting frontal obstacles based on monocular vision. As it shown in Table 2, the proposed algorithm provides more accuracy (97.4%) comparing to *SURF + Template matching* method [24] which provides 97%, and *relative distance estimation* approach [17] that provides 97.1% of accuracy.

Furthermore, the computational time of the detection algorithm is estimated around 52.4 ms. This is due to the processing ROIs of $62^{\circ }$ FOV, which leads to decrease the processing time up to 50% from 106.1 ms comparing to processing the whole $92^{\circ }$ FOV images. In addition, this computational time is estimated for the detection of 800–1200 keypoints. On the one hand, if the number of detected keypoints exceeds 6000, the computational time peaks to a maximum of 100 ms, on the other hand, if it is below 300 keypoints, then the required computation time is reduced to 30 ms.

### 5.4. Results (Obstacle Avoidance)

**Figure 17:**
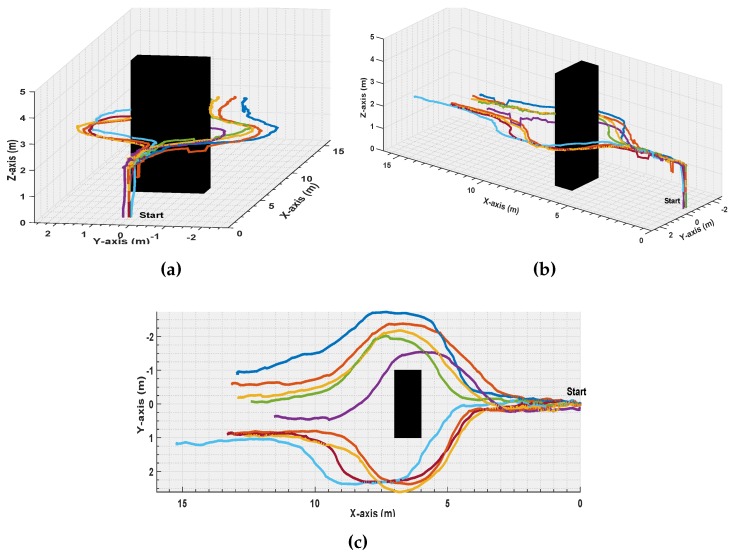
*Left-Right* Avoidance Maneuver, 9 experiments; (**a**): Front, (**b**): 3D, and (**c**): 2D.

**Figure 18:**
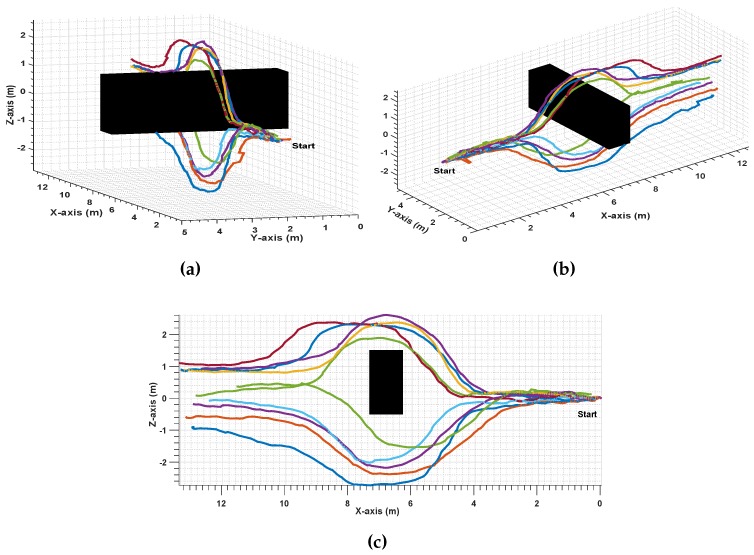
*Up-Down* Avoidance Maneuver, 10 experiments; (**a**): Front, (**b**): 3D, and (**c**): 2D.

Figure 17 and Figure 18 demonstrate an example of a set of experiments presenting the first scenario. In these experiments, the UAV is flying in a velocity of 2 m/s. All the started from the same start point, and during the the flight, an obstacle is situated in the UAV path. Figure 17 illustrates the UAV ability to perform avoidance maneuvers in the Left or Right directions of a total number of 9 experiments.

Similarly, in Figure 18, the success in avoiding hanged obstacles performing vertical maneuvers in the *z* direction by passing above and under the obstacle in a total number of 10 experiments is represented.

Finally, Table 3 shows a comparison of avoidance accuracy between two methods from the bibliography and the proposed algorithm. The accuracy results display that the best performance belongs to the proposed algorithm reaching 93% of accuracy.

## 6. Conclusions

In this paper, two algorithms have been presented as a framework to cope with cutting-edge UAVs technology. Real-time obstacle detection and avoidance is studied as a complex and essential task for intelligent aerial vehicles in transportation systems. The proposed algorithms take the advantages of onboard camera to accomplish complex tasks, that is, safe obstacle sensing and detection tasks.

The selected configuration of $62^{\circ }$ ensured the capabilities of detecting the border of an object of the size of the actual drone (58.4 × 1.3 × 54.4 ) located in the center of the image at distances higher the 15 cm, which allows to avoid obstacles, for higher obstacles or closer distances, the drone proved to be able to stop and preform hover movement, avoiding the collision. Bigger obstacles located at longer distances were avoided due to the use a high quality camera able to detect obstacle at long distances. In case of faster speeds required, the frame rate calculation and the angle should be adjusted, to allow the drone to do the calculation at proper detection. However, the change of the field of view of the camera, would only be advisable in order to allow further maneuverability in extremely dense scenarios, with short distance detection requirements which are not common in aerial scenarios where UAVs are deployed.

Keypoints used approach is based on the use of SIFT features. The performance obtained proved to be good in both computational time and overall detection performance scheme. The nature of the approach made it possible to adapt it to different set of features beyond the use of SIFT, such as the FAST-BRIEF pair [37] and BRISK [38] which proved to provide better performance in different scenarios. Future works will try to analyze the advantages of adding these sets of features to the presented approach.

The usefulness and advantages of the presented reliable solutions are demonstrated through real results under demanding circumstances, such as, complex artificial and human obstacles. Hence, complex scenarios are evaluated and difficulties are successfully overcame by means of monocular camera processing, where the relative size expansion of the obstacles are estimated and the approaching obstacles are detected from a distance between 90 and 120 cm with 97.4% of total accuracy. The various performed tests proved both, the trustable performance of the algorithms provided and the improvements in comparison to the previous works presented in literature.

The strengths of the presented applications are clearly stated in the paper, where according to the UAV path, the obstacle detection and avoidance algorithms demonstrate hight accuracy in the detection process and send maneuver commands to the UAV based on the obstacle and UAV positions. However, the specific drawbacks that should be taken into account are mainly related with the nature of the sensing devices used, that is, the monocular camera has the drawback of the high sensitivity to lighting conditions; such as direct sun light may lead to lack of information.

Future works will focus on two main lines. The tests of the application with different features, as commented. The second line is focused on the development of advanced control system, with higher capacities. These includes the use of fuzzy logic, and other advanced AI approaches.

## Figures and Tables

**Table 1:** Accuracy of Detection Algorithm.

	Indoor				Outdoor				
	People	Obstacle	Pillar	Wall	People	Obstacle	Tree	Wall	Total
Situated	200	110	80	80	200	120	140	70	1000
Detected	196	107	79	76	196	116	135	69	974
Fail	4	3	1	4	4	4	5	1	26
Accuracy (%)									
Object	98.0	97.3	98.8	95.0	98.0	96.7	96.4	98.6	97.4
Environment	97.3				97.4				

**Table 2:** Comparison of Frontal Obstacle Detection.

Algorithm	Total	Detected	Fail	Accuracy (%)
SURF + Template matching [24]	107	104	3	97
Relative distance estimation [17]	35	34	1	97.1
Proposed Algorithm	1000	974	26	97.4

**Table 3:** Comparison of Avoidance Accuracy.

Algorithm	Total	Success	Failure	Accuracy (%)
SURF + Template matching [24]	23	20	3	87
Relative Distance Estimation [17]	35	31	4	88.57
Proposed Algorithm	100	93	7	93
